# Healthcare waste management current status and potential challenges in Ethiopia: a systematic review

**DOI:** 10.1186/s13104-019-4316-y

**Published:** 2019-05-23

**Authors:** Teshiwal Deress Yazie, Mekonnen Girma Tebeje, Kasaw Adane Chufa

**Affiliations:** Unit of Quality Assurance and Laboratory Management, School of Biomedical and Laboratory Sciences, College of Medicine and Health Sciences, University of Gondar, P.O. Box 196, Gondar, Ethiopia

**Keywords:** Healthcare waste, Waste management, Healthcare facility, Ethiopia

## Abstract

**Objective:**

During the healthcare delivery process, hazardous wastes can be generated from the health facilities. Improper healthcare waste management is responsible for the transmission of more than 30 dangerous bloodborne pathogens. The aim of this systematic review was to evaluate the healthcare waste management practice and potential challenges in Ethiopia.

**Results:**

Electronic databases and direct Google search yielded 1742 articles from which 17 studies met the inclusion criteria. The proportion of hazardous waste generated in Ethiopian healthcare facilities was unacceptably high which ranged from 21 to 70%. Most studies indicated the absence of proper waste segregation practice at the source of generation. Treatment of the healthcare waste using low combustion incinerator and/or open burning and open disposal of the incinerator ash were very common. Lack of awareness from the healthcare staff, appropriate waste management utilities and enforcement from the regulatory bodies were mainly identified as a common factor shared by most of the studies. The healthcare waste management practice in Ethiopian healthcare facilities was unsatisfactory. There should be close supervision of the waste disposal process by the regulatory bodies or other stakeholders.

## Introduction

During the healthcare delivery process, healthcare facilities (HCFs) can generate wastes and by-products [1]. Currently, there are several terms used to explain the waste generated from the HCFs such as; health facility waste, clinical waste, healthcare waste, medical waste, and biomedical waste. However, healthcare waste (HCW) is most frequently used by the articles published so far [2–24]. In this study, we used the term HCW to represent the total waste generated from the HCFs.

**Fig. 1:**
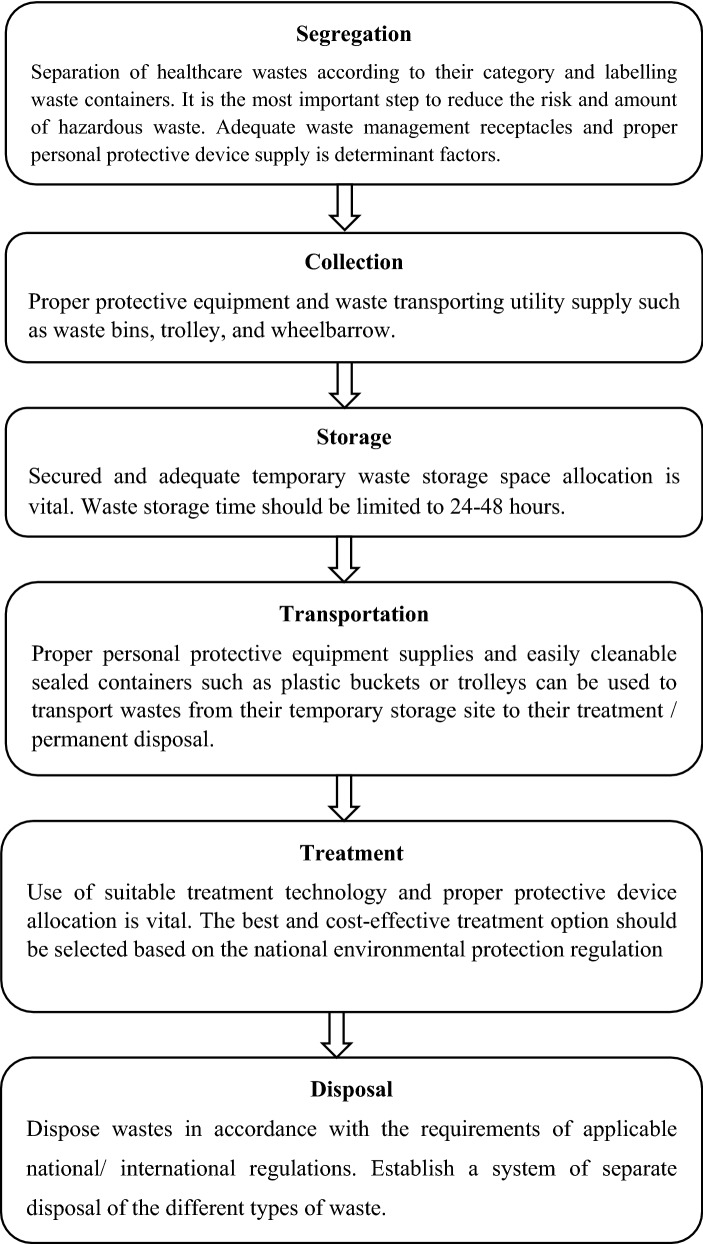
Healthcare waste management process

Healthcare waste is categorized as general and hazardous waste types [25–27]. General waste is the largest portion [26] which is originated from food preparation, administrative and housekeeping activities. Whereas, the hazardous waste is generated throughout the healthcare delivery process [19]. It includes laboratory wastes, pathological, body fluids, and sharp wastes [25, 26]. According to the guidelines, six consecutive healthcare waste management (HCWM) steps should be implemented by the HCFs [26, 28–31]. This successful management process includes segregation, collection, storage, transportation, treatment, and end up with final disposal [17, 26, 32] (Fig. 1).

Proper disposal of the HCW has become a global concern due to its public health hazards [17, 25, 33]. According to the WHO estimation, 10–25% of the HCW is hazardous [26]. However, this proportion is varied from country to country which ranged between 16 and 75% [6, 8, 12, 14, 21, 23, 34, 35]. Globally over two million healthcare workers are exposed to infections [26]. The HCW can transmit more than 30 dangerous bloodborne pathogens [36]. Poor HCWM is a problem particularly in most developing countries [5, 12, 37–40]. Several studies indicated that HCWM is still at infancy stage [3, 7, 15, 16] and particularly it is a neglected activity in Ethiopia [31]. Therefore, the aim of this systematic review was to evaluate the HCWM practice and potential challenges in Ethiopia.

## Main text

### Methods

Ethiopia is a highly populated country in Africa. During 2012 the Ethiopian population was predicted to be 84,320,987 [41]. In parallel with the rapid population growth, the number of HCFs is increasing [13, 42]. The healthcare management is categorized as primary, secondary and tertiary levels. During 2011 there were 125 hospitals, 2999 health centers, 15,668 health posts and more than 4000 private clinics in the country [43]. The health post, health center, and primary hospitals serve a population of 3000–5000, 40000 and 60,000–10,000, respectively. General and specialized hospitals serve 1 to 1.5 and 3.5–5.0 million population, respectively [44].

A qualitative research design was employed to develop this systematic review. The study was conducted according to the PRISMA (Preferred Reporting Items for Systematic Reviews and Meta-Analyses) guidelines to ensure the inclusion of relevant information in the study [45]. Studies were eligible only if they were published in the English language in peer-reviewed journals, carried out in the Ethiopian context, and accessed in the full-text format.

Published articles were searched in PubMed and Google Scholar by two investigators (TD and MG). The search strategy was employed using a combination of keywords and Boolean functions; “healthcare waste” OR “medical waste” OR “clinical waste” OR “infectious waste” OR “hospital waste” OR “healthcare facility waste” OR hazardous waste” OR “biomedical waste” OR “medical waste” AND “Ethiopia”. In addition, a direct Google search was also employed. Then, the searched articles from the two investigators were compiled together and screened for duplication. Finally, reference lists cited by each eligible study were assessed to identify additional articles. We used an endnote X9 version citation manager software to manage the citation.

Two authors (TD and KA) independently extracted data using the predefined data extraction sheet. Inconsistent data between the two data abstracters were resolved by discussion and involvement of a third co-author (KA). Data were abstracted containing first author and year of publication, setting, key findings (availability of waste management utilities/devices, waste segregation practice, hazardous waste proportion, waste treatment, final disposal, potential challenges) and additional relevant findings were paraphrased.

Before starting data extraction, selection process of the relevant studies was explained under the characteristics of included studies using texts and graphical presentation. After data extraction, the findings were grouped together into three thematic areas ‘waste generation, segregation and use of proper waste management utilities’, ‘waste treatment and disposal practices’, and ‘potential challenges. Finally, data were presented using texts.

To ensure reliability, articles were searched systematically using a combination of key terms and Boolean functions by two authors independently. The quality of the data was assured through extracting by two authors independently using the predefined data extraction checklist. Any inconsistent data from the two data extractors were resolved by discussion and involvement of a third co-author (Kasaw). In addition, the review process was done using the PRISMA guidelines.

### Results

#### Characteristics of the included studies

**Fig. 2:**
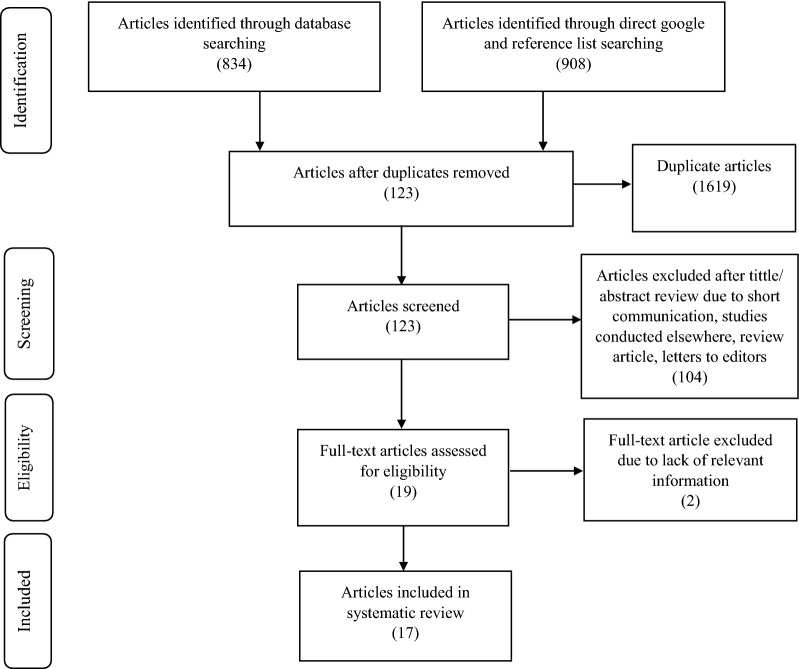
Flowchart to describe the selection of articles for the systematic review

This systematic review was conducted on published studies which were conducted among the Ethiopian healthcare facilities. An online electronic search was done using Google Scholar and PubMed databases and we identified 834 articles. In addition, from reference lists of the included studies and direct Google search, we identified 908 articles. From a total of 1742 articles, 1619 data files were removed due to duplication. Further, 123 articles were refined by their title and abstract and 104 studies were excluded due to short communication, lack of relevant data with respect to this systematic review, unpublished student thesis, studies conducted elsewhere in another country, a review article on the legal framework, and letters to editors. Nineteen full-text articles were reviewed and two of them were excluded due to lack of relevant information for this systematic review as one study was conducted on developing new models for HCWM and the other was conducted on the prevalence of injuries associated with the mismanagement of HCWs among the waste collectors. Finally, we included 17 full-text articles (Fig. 2).

#### Waste generation, segregation and use of utilities

Most studies were obtained from the central and northwest regions of Ethiopia. Studies indicated no waste segregation practice [2, 13, 14, 17, 44]. In some cases; however, there was very limited segregation [5, 6, 8, 18, 20, 21, 46]. Due to this reason, the proportion of the hazardous waste generation rate becomes unacceptably high which range from 21 to 75% [2, 6, 9, 12, 14, 17, 21, 23]. Privately owned HCFs generated a higher proportion of hazardous wastes than government-run facilities [23]. There was no use of proper color-coded bins for waste segregation [5, 9]. General and infectious wastes were mixed together [9]. Plastic buckets were used to store the HCW temporarily [17, 27]. Disinfection of waste storage and/or transporting utilities was uncommon [6, 17].

#### Waste treatment and disposal practices

Low combustion incinerator was used to treat all the HCW types [2, 8, 9, 12, 14, 21, 46]. In other cases, studies indicated the use of incineration and open burning methods to treat hazardous and general wastes, respectively [5, 6, 8, 14, 17, 20, 27]. With respect to disposal of the treated waste by-products, some studies used burial pit [2, 6] while other studies disposed of in an unsanitary way simply by open dumping [8, 12, 27].

#### Potential challenges

In the Ethiopian context, there was no separate regulation specific for the HCFs to enforce them for the proper management of the hazardous waste. Though currently they are not updated and lacked proper compliance on their implementation, there are three HCWM guidelines prepared by the Federal Ministry of Health (FMoH), Food, Medicine and Healthcare Administration and Control Authority (FMHACA), and Federal Environmental Protection Authority (FEPA) independently [29, 30, 46]. In addition, studies indicated lack of training, awareness, staff resistance, managerial poor commitment, lack of adequate resources, negligence, and unfavorable attitude of the healthcare staff were the main identified challenges [8, 10, 12–14, 46, 47]. Most studies measured the aforementioned potential factors either quantitatively or qualitatively that could affect HCFs to implement the proper waste management practices.

### Discussion

Currently, HCWM is a public health and environmental concern worldwide particularly in the developing countries [4, 48]. Hazardous waste mismanagement affects all individuals particularly healthcare providers. The general and hazardous waste types should be properly segregated at their source of generation [25, 49–51]. However, in this systematic review, studies mentioned the absence of waste segregation practices [2, 5, 12, 14, 17, 20, 21, 32, 44]. Probably this could be due to lack of the appropriate waste segregation utilities, lack of awareness or lack of enforcing laws and/or regulations. It is also a continent-wide problem by which a systematic review in the African region indicated that 47% of the studies mentioned the absence of waste segregation [52]. The proportion of hazardous HCW is varied in Ethiopia which ranged from 21 to 70% [14, 44]. This proportion is higher than the hazardous waste threshold (10–25%) predicted by the WHO [26]. In one study, even the amount of hazardous waste was higher than the general waste [14]. This could be due to the fact that during the segregation process even a very small amount of hazardous waste is added to the general waste category, then the entire mass of the general waste can be unnecessarily polluted by the hazardous waste.

The segregated HCW types are required to be collected separately using waste collecting utilities designed for each type of HCW [26, 31]; however, in this systematic review, studies indicated the aggregate collection of the different HCW types using a single container [9, 17, 21]. This could be due to the lack of awareness of the health hazards associated with the aggregate collection of all the HCW types by the waste collectors because in Ethiopian context waste collectors are mostly recruited from the low educational level and they might not provide adequate training as the healthcare professionals.

According to WHO guidelines, all hazardous HCW types generated from the HCFs should be stored in utility rooms prepared for cleaning equipment, dirty linen and waste storage [26]. If these facilities are not available, the HCW can be stored in other secured locations [31, 53]. However, studies indicated hazardous waste storage practices using the primary waste segregation containers stationed around the corridors [27, 44]. This unacceptable waste storage practice could probably due to the lack of sufficient and isolated waste storage spaces away from direct public access.

Incineration is the most widely used waste treatment method to treat hazardous HCW before the final disposal particularly in most developing countries. In Ethiopia, incineration and open burning are common treatment methods to treat hazardous and general waste types, respectively. However, incinerators are often operated under sub-optimal conditions mostly with untrained personnel [4]. Thus, due to inadequate incineration harmful substances can be released into the environment [27]. Regarding disposal of the HCW, incinerator ash is commonly disposed of in burial pit or open dumping. Most studies showed HCWM noncompliance with the requirements of the national and international guidelines [2, 5, 6, 8, 9, 12, 14, 18, 21, 27, 44]. This could be due to lack of the appropriate quantity and/or type of waste management utility supply, adequate financial allocation, specific laws, and regulations.

Healthcare waste management is a complex and challenging process and in this systematic review lack of training [2, 5], accessible guideline [2, 6, 12, 21, 46], regular supervision, appropriate utility supply, management support, and specific rules/regulations are identified as a major challenge for having effective waste management system [5, 13, 17, 23, 24, 27, 44, 46] that needs establishment of an immediate strategy to reduce the potential problems associated with the mismanagement of the hazardous wastes emanated from the healthcare establishments.

In conclusion, the HCW generation rate was high but its management very poor. Lack of accessible guideline, waste management utility, adequate training, financial constraint, and poor managerial supports were identified as the main challenges. There should be sufficient resource allocation, periodic training, and strict supervision by the stakeholders.

## Limitations

In this systematic review, liquid waste management was not considered. In addition, recycling practices of the reusable materials from the HCWs were not considered.

## Data Availability

Not applicable.

## Abbreviations

HCW: healthcare waste
HCFs: healthcare facilities
HCWM: healthcare waste management
